# Respiratory Complications after Cystectomy with Urinary Diversion: Avoidable Complications or Ineluctable Destiny?

**DOI:** 10.3390/jcm13061585

**Published:** 2024-03-10

**Authors:** Silvia Martinez Carrique, François Crettenand, Kevin Stritt, Perrine Bohner, Nuno Grilo, Sonia Rodrigues-Dias, Beat Roth, Ilaria Lucca

**Affiliations:** 1 Department of Urology, University Hospital of Lausanne, University of Lausanne, 1011 Lausanne, Switzerland; francois.crettenand@chuv.ch (F.C.); kevin.stritt@chuv.ch (K.S.); perrine.bohner@chuv.ch (P.B.); sonia.rodrigues-dias@chuv.ch (S.R.-D.); beat.roth@chuv.ch (B.R.); ilaria.lucca@chuv.ch (I.L.); 2 Department of Urology, University Hospital of Bern, University of Bern, 3010 Bern, Switzerland

**Keywords:** cystectomy, ERAS^®^, respiratory complications

## Abstract

**Background:** Cystectomy with urinary diversion (CUD) is a highly morbid surgery. Despite implementing an enhanced recovery after surgery (ERAS^®^) protocol, postoperative respiratory complications (PRC) within 30 days after surgery remain frequent. This study aims to identify patients at higher risk of developing PRC after CUD. **Methods:** We conducted a retrospective analysis of 242 patients who underwent CUD at Lausanne University Hospital from 2012 to 2022, adhering to ERAS^®^ guidelines. Data on postoperative complications, including pneumonia, respiratory failure, pulmonary embolism, lobar atelectasis, and pleural effusion, were analyzed. Chi-square and Mann–Whitney U tests compared patients with and without PRC. A multivariable Cox model identified independent prognostic factors. **Results:** PRC occurred in 41 patients (17%). Those with PRC experienced longer hospital stays and higher 30-day mortality rates. Poor ERAS^®^ compliance was a significant risk factor. Multivariable analysis showed pneumonia was associated with postoperative ileus, while pulmonary embolism correlated with infectious and cardiovascular complications. **Conclusions:** PRC result in extended hospitalization and decreased survival. Rigorous adherence to ERAS^®^ protocols, including early mobilization, respiratory physiotherapy, and avoiding nasogastric tubes, is essential for preventing PRC.

## 1. Introduction

Bladder cancer (BC) is the fourth most frequent type of cancer in men in the Western world. The risk of developing bladder cancer at 75 years of age is 2% to 4% in men and 0.5% to 1% in women [1]. The average age at diagnosis is 65–70 years. Thus, in Europe and the United States, bladder cancer accounts for 5% to 10% of all cancers in men [2]. Bladder cancer is almost 4 times more frequent in men, but the mortality rate is higher in women [3]. Radical cystectomy (RC), including lymph node dissection, is the standard treatment for muscle-invasive and recurrent high-risk non-muscle-invasive BC [4]. Simple cystectomy is a procedure performed in some patients suffering from functional urinary problems and/or neurogenic bladders in which conservative or less invasive treatments have failed. The complication rate after cystectomy with urinary diversion (CUD) is between 25% and 57% and is usually increased by unfavorable patient characteristics [5,6,7]. Mortality rates following RC are around 3% [8]. Thus, it is undisputedly clear that patients who must undergo CUD diversion benefit from Enhanced Recovery after Surgery (ERAS^®^) protocols, which have already been applied in many different surgical procedures to improve patient care and reduce complications.

Early postoperative complications (PRC) are those that occur during hospitalization or within the first 30 days after surgery.

Early postoperative respiratory complications (PRC) are frequent, influencing the length of stay (LoS) and overall survival [9]. The ERAS^®^ guidelines for cystectomies have several items that aim to reduce respiratory complications, such as early mobilization and control of fluid overload, which in turn aim to reduce other complications such as ileus [10]. In the literature, between 9 and 12% of patients are shown to present PRC after undergoing CUD [11,12]. Moreover, a study by the University of California and the University of Iowa in which more than 6500 patients were analyzed showed that those with both a respiratory complication and a postoperative infection had the highest overall odds of mortality [13]. Consequently, understanding the leading causes of PRC is of paramount importance to improve overall survival.

The aim of this study is to identify the risk factors (including adherence to the ERAS^®^ guidelines) for PRC after CUD in our uniform cohort of patients.

## 2. Materials and Methods

### 2.1. ERAS^®^ Protocol

Since 2012, all cystectomy patients operated at our tertiary center have undergone a standardized ERAS^®^ protocol as previously published [14]. A multidisciplinary team involving clinical nurses, anesthesiologists, urologists, and physiotherapists continuously reevaluates and improves perioperative ERAS^®^ principles and patient management. We employ the prospective ERAS^®^ Interactive Audit System (EIAS) (Encare AB, Stockholm, Sweden) registry to prospectively collect preoperative, intraoperative data and postoperative data as previously described [15].

### 2.2. Patient Selection

We retrospectively analyzed our prospectively maintained ERAS^®^ database comprising 242 patients who underwent cystectomy at our center between 2012 and 2022. The study included all patients who underwent oncological or non-oncological (functional and neurogenic issues) cystectomies with urinary diversion (ileal conduit, orthotopic ileal bladder substitution, heterotopic ileal catheterizable reservoir, or ureterocutaneostomy). All patients were intended to be treated according to the ERAS^®^ guidelines for cystectomies, and compliance with the ERAS^®^ protocol was assessed accordingly. No exclusion criteria were applied.

### 2.3. Data Collection

Demographic, oncologic, and perioperative variables, including respiratory complications, were retrieved from the ERAS^®^ database. All complications were verified retrospectively via a review of inpatient and outpatient medical records, including biochemical and imaging examinations. Complications were reported according to the modified Clavien–Dindo classification. Complications were also categorized according to the involved organ system. Each complication was classified according to the time of occurrence postoperatively. The World Health Organization (WHO) performance score and the American Society of Anesthesiologists (ASA) score were used to assess the patient’s physical status prior to surgery [16].

The following complications were defined as respiratory complications: lobar atelectasis, pneumonia, pleural fluid, pulmonary embolism, respiratory failure, and pneumothorax. Other postoperative complications analyzed were digestive, cardiovascular, and/or infectious complications, anastomotic leakage, and anesthesia-related complications. Postoperative ileus was defined as oral intake intolerance that persists beyond 4 days after radical cystectomy or by nausea and emesis accompanied by abdominal distention requiring gastrointestinal rest at any time postoperatively. The duration of the operation, intraoperative blood loss, intraoperative volume administration, pain management (time, dosage, and drugs used), and the length of hospital stay were also recorded. We also collected data about additional surgical intervention for CUD-specific complications during the whole follow-up (reoperations not related to CUD were excluded from this analysis).

### 2.4. Outcome Measurement

The primary endpoint was the rate of early (within 30 days) and late (after 30 days) PRC, defined as [17]:

- Respiratory failure confirmed by hypoxemic (PaO_2_ < 60 mmHg) or hypercapnic (PaCO_2_ > 500 mmHg) arterial gasometry.
- Radiologically confirmed pneumonia, lobar atelectasis, or pulmonary embolism by conventional X-ray or by computer tomography (CT) and certified by a radiologist.
- Pleural fluid or pneumothorax needing thorax drainage.

Secondary endpoints were high-grade complication rates, complication rates according to organ systems involved, reoperation rate (for cystectomy-associated complications), and factors influencing mortality.

**Figure 1:**
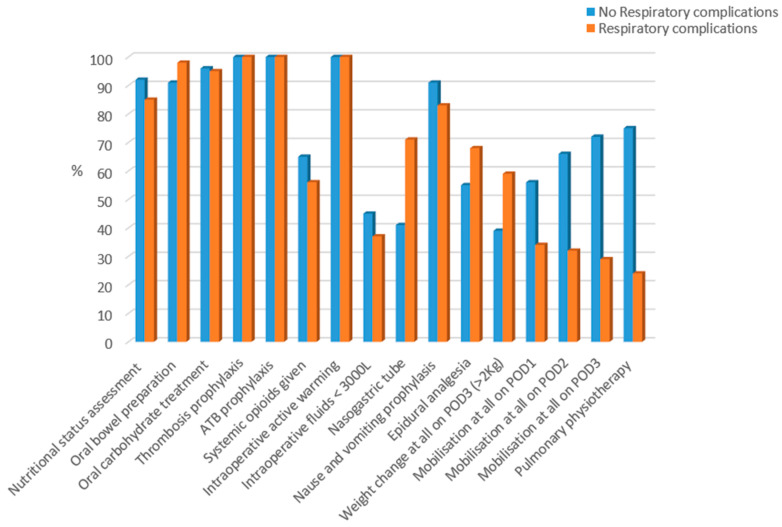
ERAS^®^ compliance in patients with and without postoperative respiratory complications.

Adherence to the ERAS^®^ program was calculated over 16 individual items [Figure 1]. The percentage of adherence was calculated as the number of patients adhering to the items divided by the total number of patients in each group [18].

### 2.5. Statistical Analysis

Categorical variables are presented as numbers and proportions, and continuous variables as median and interquartile range (IQR). To compare patients with respiratory complications within 30 days after surgery to those without (control group), Chi-square and Mann–Whitney U tests were used for categorical and continuous variables, respectively. Statistical tests were two-sided, and a *p* value < 0.05 was considered statistically significant. A multivariable Cox proportional hazards model was fitted to identify independent, significant prognostic factors. Statistical analyses were performed with STATA 17 (College Station, TX, USA) and R version 4.0.3 (10 October 2020).

## 3. Results

**Figure 2:**
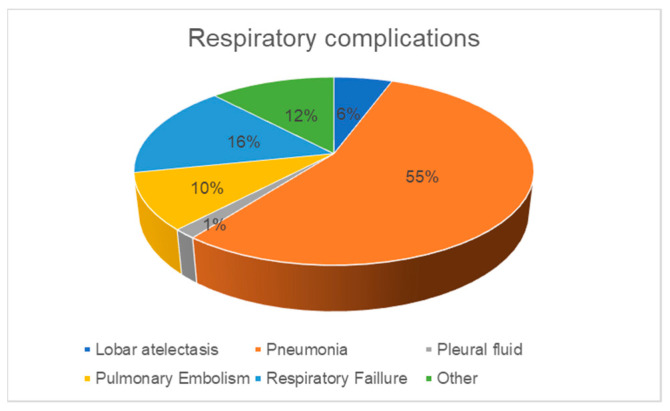
Respiratory complications.

A cohort of 242 patients was included in the analysis, comprising 179 (74%) males and 63 (26%) females. The average patient age was 72 yrs. Eight (3%) of the patients had pre-existing severe lung disease at the time of cystectomy. Within 30 days following surgery, 41 patients (17%) experienced postoperative respiratory complications, including pneumonia, respiratory failure, pulmonary embolism, lobar atelectasis, and pleural effusion [Figure 2].

There were no significant differences observed between the groups with vs. without respiratory complications in terms of sociodemographic characteristics. Among all patients, 98 (40%) were smokers; 13% of them experienced respiratory complications. The mean body mass index (BMI) was 26.2 for both groups. Most patients (n = 213; 88%) underwent radical cystectomy for bladder cancer, while 25 (10%) underwent the procedure for functional reasons and 4 (2%) for cancer of adjacent organs. Notably, the latter two groups did not exhibit any respiratory complications. Preoperative chemotherapy was administered to 49 (20%) patients. The most common urinary diversion procedure performed was ileal conduit (78%), followed by orthotopic neobladder (15%) and ureterocutaneostomy (6%) [Table 1].

Significant differences were observed between the two groups in terms of postoperative mobilization: the median time to mobilization in the group with respiratory complications was 2 days, whereas it was 1 day in the group without complications (*p* = 0.01). On postoperative day (POD) 1, 56% of patients without respiratory complications were mobilized, whereas it was only 34% in the group with respiratory complications (*p* = 0.009). On POD 2 and POD 3, mobilization rates were 66% vs. 32% (*p* < 0.001) and 72% vs. 29% (*p* = 0.001), respectively, for the two groups.

Weight gain was identified as a significant factor, as patients with respiratory complications had a higher gain of weight within the first 3 days postoperatively (2 kg vs. 1 kg, *p* = 0.004). Also, the occurrence of weight gain exceeding 2 kg during the initial three postoperative days was significantly higher in the group of patients who encountered respiratory complications, in contrast to the group without such complications, with proportions of 59% and 39%, respectively (*p* = 0.02).

In line with previous studies and as a logical consequence [13], patients with respiratory complications had a longer length of hospital stay (LoS), with a median of 25 days compared to 14 days in the non-complication group (*p* < 0.001). Additionally, the mortality rate at 30 days postoperative was significantly higher in patients with respiratory complications (20% vs. 2%, *p* < 0.001) [Table 2].

Among the cohort of 41 patients who experienced respiratory complications, 32 (78%) had a preceding ileus occurring at an average of 3 postoperative days (*p* < 0.001). At multivariable analysis, respiratory complications were independently associated with postoperative ileus (odds ratio [OR] 5.72, 95% confidence interval [CI] 2.45–13.35, *p* < 0.001) and renal complications (OR 3.73, CI 1.46–9.52, *p* = 0.006). In line with the ileus data, significant differences were found in both groups regarding the avoidance of postoperative nasogastric tubes: in patients without respiratory complications, nasogastric tubes could be avoided in 59% of them, but only 29% of patients with respiratory complications did not have a tube, compared to 71% who did (*p* < 0.001).

Blood loss was only significant in the univariate but not the multivariate analysis [Table 3].

In terms of respiratory complications, pneumonia showed a significant association with postoperative ileus (OR 6.51, CI 2.35–18.10, *p* < 0.001). On the other hand, pulmonary embolism was significantly linked to infectious complications (OR 15.56, CI 2.67–90.40, *p* = 0.002) as well as cardiovascular complications (OR 6.10, CI 1.03–36.32, *p* = 0.05) [Table 4 and Table 5].

Overall adherence to the ERAS^®^ protocol was high (>65%). Respiratory physiotherapy emerges as a mitigating factor against the development of respiratory complications. Within our study population, a mere 24% of patients who encountered respiratory complications had undergone respiratory physiotherapy starting from the initial postoperative day. In stark contrast, 75% of patients without respiratory complications received this preventative treatment in adherence to ERAS^®^ guidelines (*p* < 0.001) [Figure 1].

**Figure 3:**
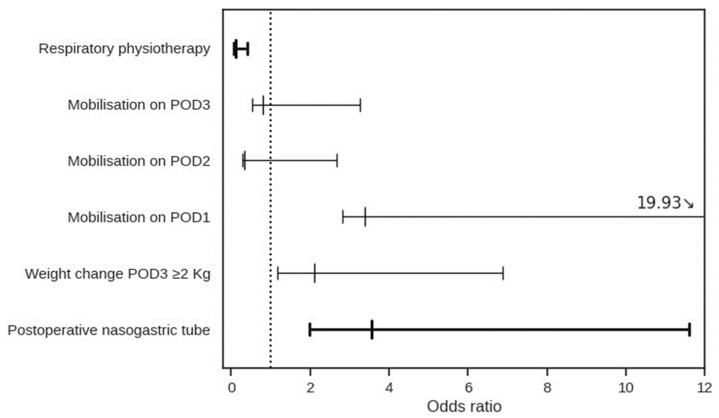
Multivariable analysis of ERAS^®^ protocol.

Among the items in the ERAS^®^ guidelines, only respiratory physiotherapy (OR 0.12, CI 0.05–0.30, *p* < 0.001) had a significant association with fewer respiratory complications at multivariate analysis influence. On the other hand, the use of a nasogastric tube was shown to be detrimental (OR 3.55, CI 1.57–8.10, *p* = 0.002) [Figure 3].

## 4. Discussion

The incidence of respiratory complications after major abdominal surgery is 23% [19]. In particular, respiratory complications are common after CUD even when pre-, intra-, and postoperative ERAS^®^ recommendations are followed. Many studies in recent years have described non-modifiable risk factors for respiratory complications, such as age, sex, or ASA >II preoperative status [20,21,22]. In our population, however, preoperative factors were not significantly associated with respiratory complications. Adherence to an ERAS^®^ protocol, however, was essential in the postoperative period and might prevent postoperative respiratory complications. Notably, no respiratory complications were found in patients undergoing surgery for functional disorders (N = 25). This might be associated with a very small sub-population and a shorter intervention time, although further investigation is needed.

In several studies, pneumonia represents the main respiratory complication after major surgery (up to 10% of patients) [23], while respiratory failure was the second most frequent type of respiratory complication [24]. This is in line with our data showing that 68% and 18% of the total 17% of respiratory complications were due to pneumonia and respiratory failure, respectively. As a consequence, many studies show that respiratory complications represent a considerable increase in hospital stay, health costs, and 30-day postoperative mortality [25]. The mechanisms that can lead to pneumonia after major abdominal surgery include poor expectoration due to immobility and pain, as well as bronchial aspiration of gastric contents in the presence of vomiting or a nasogastric tube. Ileus is a frequent complication after RC, given the use of the ileum for urinary diversion, and pneumonia is—as confirmed in our cohort—related to ileus. In turn, insertion of a nasogastric tube is often performed when ileus occurs. In a randomized prospective controlled trial, postoperative hospitalization, narcotic analgesic administration duration, flatus, defecation, and nasogastric tube termination time were shorter in patients with early mobilization after radical cystectomy [26]. In our study cohort, we could observe significant differences in early mobilization, postoperative weight gain, and in the application of early respiratory physiotherapy measures between the two groups. This suggests that the ERAS^®^ protocol was not followed in the same way in all patients, as this protocol recommends early mobilization and respiratory physiotherapy measures from the first postoperative day as well. Our results are consistent with those obtained for colorectal surgery in our center, which showed a higher number of respiratory complications in patients who were less adherent to the ERAS^®^ protocol [27]. This confirms the importance of following the ERAS^®^ protocol whenever possible to decrease morbidity and mortality after CUD.

The 3-day postoperative weight gain was significantly higher in patients with respiratory complications in our study and complies with recent literature. As a consequence, recent publications suggest aiming for “zero” intraoperative fluid balance, with early oral fluid intake to avoid unnecessary postoperative intravenous infusions [28]. This is also recommended in the ERAS^®^ protocol for radical cystectomy.

Length of stay also correlates with pulmonary complications. Presumably, length of stay is a confounder variable for the significant association between other complications and respiratory complications. Whether respiratory complications may have influenced the occurrence of other complications leading to a longer hospitalization, or vice versa, is, however, not always possible to determine exactly.

Our study does not come without possible limitations. For example, the retrospective analysis rendered it sometimes difficult to determine beyond doubt whether the adherence to the ERAS^®^ protocol was accurate, even if most parameters were prospectively assessed in the ERAS^®^ database. According to the ERAS^®^ guidelines, it is recommended that patients limit their total water consumption to no more than 1 L on POD1, with an increase to 2 L on POD2. The literature preceding our investigation has established a positive correlation between the equilibrium of salt and water intake and an increase in body weight, which could be as significant as 3 kg [29]. Given the pivotal role of weight gain within the first three days post-surgery in our analysis, we cannot discount the possibility of a more pronounced association between the regulation of salt and water intake and the incidence of respiratory complications. Unfortunately, in our study, we had no information if the total hydration was oral or intravenous. This underscores the need for further research to elucidate the impact of postoperative fluid management on patient outcomes, particularly in the context of respiratory health.

Indeed, a prospective trial precisely assessing the adherence to such an ERAS^®^ protocol would allow us to better assess each patient’s adherence to the ERAS^®^ protocol in “real time”. Furthermore, other variables could have been better studied, such as detailed smoking habits, which in our study were not found to be related to respiratory complications. Still, our database is consistent and represents a consecutive cohort of cystectomy patients over a period of 10 years.

Another limitation is the fact that ERAS^®^ is a fluid and constantly changing and improving tool, which makes a statistical analysis of sometimes changing factors difficult; thus, statistical analysis might not always be fully representative. Improving the quality of preoperative information about key aspects of the protocol may be a fruitful way to improve patients’ adherence to ERAS^®^ guidelines.

## 5. Conclusions

Respiratory complications are major complications that negatively influence the length of stay and mortality. The use of a nasogastric tube, the delay in postoperative mobilization, and excessive weight gain were all associated with respiratory complications in our study. Interestingly, ERAS^®^ guidelines for cystectomy and urinary diversion contain items designed to address the aforementioned risk factors. The risk of complications is increased with this type of condition, which has certain risk factors. Consequently, adequate adherence to an ERAS^®^ protocol in pre-, intra-, and postoperative patient management seems to be of the utmost importance to avoid potentially preventable complications, in particular when it comes to major morbid procedures such as cystectomy with urinary diversion.

## Figures and Tables

**Table 1:** Patient demographic characteristics.

Variable	All Patients (n = 242)	Respiratory Complications		*p*
		No (n = 201)	Yes (n = 41)	
Age-median (IQR)	72 (64–77)	72 (63–77)	72 (64–76)	0.72
Gender-n (%)				0.21
Female	63 (26)	56 (28)	7 (17)	
Male	179 (74)	145 (72)	34 (83)	
Smoking-n (%)	98 (40)	78 (39)	20 (49)	0.31
Alcohol-n (%)	43 (18)	35 (17)	8 (20)	0.92
BMI-median (IQR)	26 (23–29)	26 (23–29)	26 (22–27)	0.29
Diabetes-n (%)	46 (19)	32 (16)	9 (22)	0.76
WHO performance score ≥ 2-n (%)	16 (7)	13 (6)	3 (7)	0.66
Preoperative chemotherapy-n (%)	49 (20)	38 (19)	11 (27)	0.35
Previous abdominal surgery-n (%)	94 (39)	81 (40)	13 (32)	0.39
Underlying disease-n (%)				
Severe heart disease	23 (10)	18 (9)	5 (12)	0.72
Severe pulmonary disease	8 (3)	5 (2)	3 (7)	0.27
ASA class-n (%)				0.11
I	3 (1)	3 (1)	0 (0)	
II	135 (56)	112 (56)	23 (56)	
III	101 (42)	85 (42)	16 (39)	
IV	3 (1)	1 (1)	2 (5)	
Urothelial Carcinoma-n (%)	213 (88)	172 (86)	41 (100)	0.02
pT stage-n (%)				0.29
pT0	17 (8)	12 (6)	5 (12)	
pTis	24 (11)	17 (8)	7 (17)	
pTa	9 (4)	9 (4)	0 (0)	
pT1	17 (8)	13 (6)	4 (10)	
pT2	36 (17)	32 (16)	5 (12)	
pT3	74 (35)	65 (32)	11 (27)	
pT4	36 (17)	27 (13)	9 (22)	
pN stage-n (%)				0.19
pN0	135 (63)	113 (66)	22 (54)	
pN1	22 (10)	19 (11)	3 (7)	
pN2	30 (14)	21 (12)	9 (22)	
pN3	3 (1)	3 (2)	0 (0)	
pNX	23 (11)	16 (9)	7 (17)	
Functional disorder-n (%)	25 (10)	25 (12)	0 (0)	0.04
Other malignancy-n (%)	4 (2)	4 (2)	0 (0)	0.81

BMI: body mass index, ASA: American Society of Anesthesiology, WHO: World Health Organization.

**Table 2:** Surgical and postoperative related parameters.

Variable	All Patients (n = 242)	Respiratory Complications		*p*
		No (n = 201)	Yes (n = 41)	
Surgical procedure				
Bricker Ileal conduit-n (%)	189 (78)	156 (78)	33 (81)	0.84
Cutaneous ureterostomy-n (%)	15 (6)	14 (7)	1 (2)	0.46
Orthotopic neobladder-n (%)	38 (16)	31 (15)	7 (17)	0.98
Operation duration (min) median-(IQR)	383 (327–450)	380 (330–450)	401 (327–446)	0.48
Intraoperative blood loss (mL) median-(IQR)	400 (300–787)	400 (275–700)	700 (350–1000)	0.04 *
Intraoperative fluids (mL) median-(IQR)	3500 (2500–4500)	3500 (2500–4500)	3500 (3000–4540)	0.17
Infectious complications-n (%)	67 (28)	38 (19)	29 (71)	0.02
Cardiovascular complications-n (%)	66 (27)	49 (24)	17 (41)	0.04
Renal complications-n (%)	34 (14)	21 (10)	13 (32)	<0.001
Ileus-n (%)	110 (45)	87 (43)	23 (56)	<0.001
LOS-median (IQR)	15 (12–21)	14 (11–19)	25 (19–48)	<0.001
POD mobilization-median (IQR)	1 (1–2)	1 (1–2)	2 (1–2)	0.01
Weight gain (kg) *-median (IQR)	1 (1–2)	1 (1–2)	2 (1–3)	0.004
Readmission ** rate-n (%)	37(15)	33 (89)	4 (11)	0.39

* at 3 days. ** at 30 days. POD: postoperative day. LOS: length of stay.

**Table 3:** Univariable and multivariable analysis of all respiratory complications.

Variable	Univariate			Multivariate		
	OR	95% CI	*p*	OR	95% CI	*p*
Age	1.01	0.97–1.04	0.73			
BMI	0.96		0.13			
Smoker	1.50	0.76–2.95	0.24			
Severe pulmonary disease	3.10	0.71–13.50	0.13			
WHO score	1.22	0.77–1.93	0.40			
ASA score	1.26	0.68–2.34	0.45			
Blood loss	1.01	1.00–1.01	0.03	1.00	0.99–1.00	0.09
Preoperative chemotherapy	1.57	0.73–3.42	0.25			
Length of operation	1.00	0.99–1.01	0.45			
Tumor stage	0.93	0.74–1.15	0.50			
Time to oral pain control	1.04	1.01–1.08	0.03	1.02	0.98–1.06	0.41
Renal complications	3.98	1.79–8.84	0.001	3.73	1.46–9.52	0.006
Cardiovascular complications	2.20	1.09–4.42	0.03	1.55	0.69–3.44	0.28
Ileus	5.61	2.54–12.38	<0.001	5.72	2.45–13.35	<0.001

**Table 4:** Univariable and multivariable analysis of pulmonary embolism.

Variable	Univariate			Multivariate		
	OR	95% CI	*p*	OR	95% CI	*p*
Age	1.02	0.94–1.10	0.66			
BMI	0.98	0.83–1.16	0.85			
Smoker	1.08	0.24–4.96	0.91			
Severe pulmonary disease	-					
WHO score	0.48	0.13–1.85	0.29			
ASA score	0.60	0.14–2.58	0.49			
Blood loss	1.01	0.99–1.00	0.09			
Preoperative chemotherapy	1.60	0.30–8.51	0.58			
Length of operation	1.01	1.01–1.02	0.03	1.01	0.99–1.01	0.07
Tumor stage	0.99	0.61–1.61	0.97			
Time to oral pain control	0.89	0.67–1.20	0.45			
Renal complications	2.47	0.46–13.30	0.29			
Infectious complications	14.92	2.77–80.13	0.002	15.56	2.67–90.40	0.002
Cardiovascular complications	7.08	1.33–37.45	0.02	6.10	1.03–36.32	0.05
Ileus	0.19	0.02–1.59	0.12			

**Table 5:** Univariable and multivariable analysis of pneumonia.

Variable	Univariate			Multivariate		
	OR	95% CI	*p*	OR	95% CI	*p*
Age	0.99		0.60			
BMI	0.98		0.59			
Smoker	1.68		0.19			
Severe pulmonary disease	2.55		0.26			
WHO score	1.04		0.87			
ASA score	1.07		0.84			
Blood loss	1.00		0.32			
Preoperative chemotherapy	1.29		0.58			
Length of operation	1.01		0.78			
Tumor stage	0.98		0.91			
Time to oral pain control	1.05	1.01–1.09	0.01	1.03	0.98–1.08	0.17
Renal complications	2.74	1.10–6.82	0.03	2.54	0.92–7.03	0.07
Cardiovascular complications	1.76	0.78–3.95	0.17			
Ileus	7.09	2.60–19.30	<0.001	6.51	2.35–18.10	<0.001

## Data Availability

The datasets generated during and/or analyzed during the current study are available from the corresponding author on reasonable request.
